# Supplementary material for: Modeling glioblastoma heterogeneity as a dynamic network of cell states
Source: Mol Syst Biol. 2021 Sep 16;17(9):e10105. doi: 10.15252/msb.202010105 (PMC8444284; doi:10.15252/msb.202010105)
Supplement: Supplementary file 5 — Source Data for Figure 3 [file MSB-17-e10105-s001.zip › Figure3A_sourcedata/GSEA_3065/hallmarks_state3.GseaPreranked.1623416274744/HALLMARK_HEME_METABOLISM.html]

Details for gene set HALLMARK\_HEME\_METABOLISM[GSEA]

|  || Dataset | state3 |
| Phenotype | NoPhenotypeAvailable |
| Upregulated in class | na\_neg |
| GeneSet | HALLMARK\_HEME\_METABOLISM |
| Enrichment Score (ES) | -0.25172123 |
| Normalized Enrichment Score (NES) | -0.93349826 |
| Nominal p-value | 0.6064815 |
| FDR q-value | 0.7859195 |
| FWER p-Value | 1.0 |
Table: GSEA Results Summary

  

Fig 1: Enrichment plot: HALLMARK\_HEME\_METABOLISM      
 Profile of the Running ES Score & Positions of GeneSet Members on the Rank Ordered List

  

| PROBE | GENE SYMBOL | GENE\_TITLE | RANK IN GENE LIST | RANK METRIC SCORE | RUNNING ES | CORE ENRICHMENT || 1 | BSG |  |  | 98 | 0.347 | 0.0201 | No |
| 2 | LAMP2 |  |  | 154 | 0.309 | 0.0416 | No |
| 3 | CLCN3 |  |  | 251 | 0.263 | 0.0544 | No |
| 4 | CTSB |  |  | 392 | 0.219 | 0.0580 | No |
| 5 | SLC25A37 |  |  | 500 | 0.199 | 0.0636 | No |
| 6 | TOP1 |  |  | 501 | 0.199 | 0.0816 | No |
| 7 | MINPP1 |  |  | 536 | 0.194 | 0.0952 | No |
| 8 | ATP6V0A1 |  |  | 562 | 0.191 | 0.1096 | No |
| 9 | SLC2A1 |  |  | 587 | 0.188 | 0.1238 | No |
| 10 | ENDOD1 |  |  | 623 | 0.184 | 0.1364 | No |
| 11 | PIGQ |  |  | 658 | 0.177 | 0.1485 | No |
| 12 | RBM5 |  |  | 672 | 0.175 | 0.1629 | No |
| 13 | NFE2L1 |  |  | 709 | 0.169 | 0.1740 | No |
| 14 | GMPS |  |  | 954 | 0.142 | 0.1586 | No |
| 15 | DAAM1 |  |  | 1211 | 0.121 | 0.1399 | No |
| 16 | TFRC |  |  | 1304 | 0.115 | 0.1396 | No |
| 17 | TNRC6B |  |  | 1326 | 0.114 | 0.1475 | No |
| 18 | TSPAN5 |  |  | 1466 | 0.105 | 0.1409 | No |
| 19 | EPB41 |  |  | 1504 | 0.103 | 0.1459 | No |
| 20 | RNF19A |  |  | 1530 | 0.101 | 0.1522 | No |
| 21 | NNT |  |  | 1657 | 0.094 | 0.1461 | No |
| 22 | LPIN2 |  |  | 1687 | 0.093 | 0.1511 | No |
| 23 | USP15 |  |  | 1689 | 0.093 | 0.1594 | No |
| 24 | RIOK3 |  |  | 1799 | 0.087 | 0.1546 | No |
| 25 | MBOAT2 |  |  | 1800 | 0.087 | 0.1625 | No |
| 26 | SLC11A2 |  |  | 1941 | 0.079 | 0.1534 | No |
| 27 | CPOX |  |  | 2044 | 0.074 | 0.1483 | No |
| 28 | AGPAT4 |  |  | 2196 | 0.069 | 0.1370 | No |
| 29 | MARK3 |  |  | 2328 | 0.063 | 0.1276 | No |
| 30 | LRP10 |  |  | 2436 | 0.059 | 0.1206 | No |
| 31 | KLF3 |  |  | 2479 | 0.058 | 0.1209 | No |
| 32 | OSBP2 |  |  | 2592 | 0.054 | 0.1128 | No |
| 33 | BCAM |  |  | 2669 | 0.051 | 0.1086 | No |
| 34 | SLC7A11 |  |  | 2682 | 0.051 | 0.1118 | No |
| 35 | BMP2K |  |  | 2855 | 0.045 | 0.0960 | No |
| 36 | ADD1 |  |  | 3082 | 0.039 | 0.0734 | No |
| 37 | FBXO34 |  |  | 3113 | 0.038 | 0.0733 | No |
| 38 | GAPVD1 |  |  | 3354 | 0.032 | 0.0484 | No |
| 39 | CDC27 |  |  | 3508 | 0.028 | 0.0332 | No |
| 40 | NUDT4 |  |  | 3513 | 0.028 | 0.0353 | No |
| 41 | DCUN1D1 |  |  | 3632 | 0.025 | 0.0239 | No |
| 42 | TFDP2 |  |  | 3647 | 0.024 | 0.0245 | No |
| 43 | XPO7 |  |  | 3662 | 0.024 | 0.0250 | No |
| 44 | PPOX |  |  | 3961 | 0.016 | -0.0081 | No |
| 45 | GCLM |  |  | 4026 | 0.014 | -0.0141 | No |
| 46 | BTG2 |  |  | 4070 | 0.013 | -0.0179 | No |
| 47 | NCOA4 |  |  | 4078 | 0.013 | -0.0175 | No |
| 48 | VEZF1 |  |  | 4169 | 0.011 | -0.0270 | No |
| 49 | TMEM9B |  |  | 4192 | 0.010 | -0.0287 | No |
| 50 | ASNS |  |  | 4228 | 0.009 | -0.0319 | No |
| 51 | CIR1 |  |  | 4273 | 0.008 | -0.0363 | No |
| 52 | MAP2K3 |  |  | 4277 | 0.007 | -0.0360 | No |
| 53 | BACH1 |  |  | 4296 | 0.007 | -0.0375 | No |
| 54 | ARHGEF12 |  |  | 4401 | 0.004 | -0.0491 | No |
| 55 | EZH1 |  |  | 4440 | 0.003 | -0.0533 | No |
| 56 | P4HA2 |  |  | 4511 | 0.001 | -0.0613 | No |
| 57 | MKRN1 |  |  | 4738 | -0.004 | -0.0871 | No |
| 58 | DCAF11 |  |  | 4806 | -0.006 | -0.0943 | No |
| 59 | PICALM |  |  | 4882 | -0.008 | -0.1023 | No |
| 60 | FOXO3 |  |  | 4960 | -0.010 | -0.1103 | No |
| 61 | HEBP1 |  |  | 5022 | -0.012 | -0.1163 | No |
| 62 | GDE1 |  |  | 5136 | -0.014 | -0.1281 | No |
| 63 | FECH |  |  | 5148 | -0.014 | -0.1281 | No |
| 64 | ADD2 |  |  | 5219 | -0.016 | -0.1347 | No |
| 65 | CA2 |  |  | 5274 | -0.018 | -0.1394 | No |
| 66 | BLVRA |  |  | 5364 | -0.020 | -0.1479 | No |
| 67 | MGST3 |  |  | 5395 | -0.021 | -0.1494 | No |
| 68 | MOSPD1 |  |  | 5411 | -0.021 | -0.1493 | No |
| 69 | CAT |  |  | 5510 | -0.024 | -0.1584 | No |
| 70 | MXI1 |  |  | 5656 | -0.029 | -0.1726 | No |
| 71 | NR3C1 |  |  | 5724 | -0.030 | -0.1776 | No |
| 72 | SLC6A8 |  |  | 5778 | -0.032 | -0.1808 | No |
| 73 | BTRC |  |  | 5840 | -0.034 | -0.1848 | No |
| 74 | ALAD |  |  | 5878 | -0.035 | -0.1859 | No |
| 75 | DCAF10 |  |  | 5944 | -0.037 | -0.1901 | No |
| 76 | IGSF3 |  |  | 6015 | -0.040 | -0.1946 | No |
| 77 | NARF |  |  | 6024 | -0.040 | -0.1918 | No |
| 78 | ALDH6A1 |  |  | 6109 | -0.043 | -0.1976 | No |
| 79 | PSMD9 |  |  | 6142 | -0.044 | -0.1973 | No |
| 80 | PPP2R5B |  |  | 6240 | -0.048 | -0.2042 | No |
| 81 | SEC14L1 |  |  | 6272 | -0.049 | -0.2034 | No |
| 82 | FBXO7 |  |  | 6283 | -0.049 | -0.2001 | No |
| 83 | TRAK2 |  |  | 6284 | -0.049 | -0.1956 | No |
| 84 | SLC10A3 |  |  | 6384 | -0.052 | -0.2024 | No |
| 85 | CCDC28A |  |  | 6462 | -0.055 | -0.2063 | No |
| 86 | SLC25A38 |  |  | 6494 | -0.056 | -0.2048 | No |
| 87 | RBM38 |  |  | 6783 | -0.070 | -0.2318 | No |
| 88 | EIF2AK1 |  |  | 6796 | -0.071 | -0.2268 | No |
| 89 | CCND3 |  |  | 6959 | -0.079 | -0.2384 | No |
| 90 | KAT2B |  |  | 7042 | -0.084 | -0.2404 | No |
| 91 | UROD |  |  | 7141 | -0.090 | -0.2436 | Yes |
| 92 | SMOX |  |  | 7160 | -0.091 | -0.2375 | Yes |
| 93 | HMBS |  |  | 7199 | -0.093 | -0.2334 | Yes |
| 94 | MFHAS1 |  |  | 7281 | -0.099 | -0.2338 | Yes |
| 95 | HAGH |  |  | 7298 | -0.100 | -0.2266 | Yes |
| 96 | OPTN |  |  | 7409 | -0.109 | -0.2295 | Yes |
| 97 | SDCBP |  |  | 7454 | -0.113 | -0.2244 | Yes |
| 98 | BLVRB |  |  | 7460 | -0.113 | -0.2147 | Yes |
| 99 | HTRA2 |  |  | 7533 | -0.121 | -0.2122 | Yes |
| 100 | YPEL5 |  |  | 7616 | -0.129 | -0.2100 | Yes |
| 101 | FBXO9 |  |  | 7691 | -0.138 | -0.2060 | Yes |
| 102 | CAST |  |  | 7828 | -0.155 | -0.2077 | Yes |
| 103 | PRDX2 |  |  | 7855 | -0.159 | -0.1963 | Yes |
| 104 | UCP2 |  |  | 7873 | -0.162 | -0.1836 | Yes |
| 105 | UBAC1 |  |  | 7942 | -0.175 | -0.1756 | Yes |
| 106 | BNIP3L |  |  | 8044 | -0.192 | -0.1699 | Yes |
| 107 | GYPC |  |  | 8067 | -0.197 | -0.1546 | Yes |
| 108 | BPGM |  |  | 8071 | -0.197 | -0.1371 | Yes |
| 109 | ADIPOR1 |  |  | 8076 | -0.198 | -0.1196 | Yes |
| 110 | UROS |  |  | 8099 | -0.203 | -0.1037 | Yes |
| 111 | TCEA1 |  |  | 8230 | -0.235 | -0.0975 | Yes |
| 112 | PGLS |  |  | 8264 | -0.244 | -0.0792 | Yes |
| 113 | HDGF |  |  | 8267 | -0.246 | -0.0572 | Yes |
| 114 | ARL2BP |  |  | 8271 | -0.246 | -0.0352 | Yes |
| 115 | ISCA1 |  |  | 8330 | -0.265 | -0.0180 | Yes |
| 116 | GLRX5 |  |  | 8421 | -0.302 | -0.0011 | Yes |
| 117 | RAD23A |  |  | 8628 | -0.433 | 0.0142 | Yes |
Table: GSEA details [plain text format]

  

Fig 2: HALLMARK\_HEME\_METABOLISM: Random ES distribution      
 Gene set null distribution of ES for **HALLMARK\_HEME\_METABOLISM**
